# The Effects of Tai Chi and Qigong on Immune Responses: A Systematic Review and Meta-Analysis

**DOI:** 10.3390/medicines7070039

**Published:** 2020-06-30

**Authors:** Byeongsang Oh, Kyeore Bae, Gillian Lamoury, Thomas Eade, Frances Boyle, Brian Corless, Stephen Clarke, Albert Yeung, David Rosenthal, Lidia Schapira, Michael Back

**Affiliations:** 1 Northern Sydney Cancer Centre, Royal North Shore Hospital, St Leonards NSW 2065 Australia; kyeorebae@gmail.com (K.B.); Gillian.Lamoury@health.nsw.gov.au (G.L.); Thomas.Eade@health.nsw.gov.au (T.E.); bcorless@shoalhaven.net.au (B.C.); stephen.clarke@sydney.edu.au (S.C.); michael.back@health.nsw.gov.au (M.B.); 2 The Mater Hospital, North Sydney NSW 2060, Australia; franb@bigpond.net.au; 3 Sydney Medical School, University of Sydney, Sydney NSW 2060, Australia; 4 Center for Immunity and Pain, Kwanghye Hospital, Seoul 06174, Korea; 5 Harvard Medical School, Boston, MA 02115, USA; AYEUNG@mgh.harvard.edu (A.Y.); drose@huhs.harvard.edu (D.R.); 6 Stanford University School of Medicine, Stanford, CA 94305, USA; schapira@stanford.edu

**Keywords:** Tai Chi, qigong, immune system, immunity, inflammation

## Abstract

**Background:** Effective preventative health interventions are essential to maintain well-being among healthcare professionals and the public, especially during times of health crises. Several studies have suggested that Tai Chi and Qigong (TQ) have positive impacts on the immune system and its response to inflammation. The aim of this review is to evaluate the current evidence of the effects of TQ on these parameters. **Methods:** Electronic searches were conducted on databases (Medline, PubMed, Embase and ScienceDirect). Searches were performed using the following keywords: “Tai Chi or Qigong” and “immune system, immune function, immunity, Immun*, inflammation and cytokines”. Studies published as full-text randomized controlled trials (RCTs) in English were included. Estimates of change in the levels of immune cells and inflammatory biomarkers were pooled using a random-effects meta-analysis where randomised comparisons were available for TQ versus active controls and TQ versus non-active controls. **Results:** Nineteen RCTs were selected for review with a total of 1686 participants and a range of 32 to 252 participants within the studies. Overall, a random-effects meta-analysis found that, compared with control conditions, TQ has a significant small effect of increasing the levels of immune cells (SMD, 0.28; 95% CI, 0.13 to 0.43, *p* = 0.00), I^2^ = 45%, but not a significant effect on reducing the levels of inflammation (SMD, −0.15; 95% CI, −0.39 to 0.09, *p* = 0.21), I^2^ = 85%, as measured by the systemic inflammation biomarker C-reactive protein (CRP) and cell mediated biomarker cytokines. This difference in results is due to the bidirectional regulation of cytokines. An overall risk of bias assessment found three RCTs with a low risk of bias, six RCTs with some concerns of bias, and ten RCTs with a high risk of bias. **Conclusions:** Current evidence indicates that practising TQ has a physiologic impact on immune system functioning and inflammatory responses. Rigorous studies are needed to guide clinical guidelines and harness the power of TQ to promote health and wellbeing.

## 1. Introduction

The effectiveness of the human immune system to prevent disease and aid recovery is critical [1]. Inflammation is an adaptive biological response of the immune system that can be triggered by several factors, such as pathogens, damaged cells and toxic compounds [2,3]. In response to infection, immune cells produce pro-inflammatory cytokines [4] and suppress anti-inflammatory genes [5] as key elements of the pathogenic defense process. In the current COVID-19 pandemic, humanity is facing one of its greatest public health challenges with more physical and psychological demands placed on the immune system and a greater need for evidence-based healthy lifestyle interventions, such as increased physical activity, to support and maintain the integrity of immune functions [6]. Several studies have reported that older people with comorbid conditions are more likely to have more severe symptoms and a higher risk of mortality from COVID-19 infection compared to children or younger adults [7,8,9]. Older adults with chronic disease and diminished immune responses have been found to have an increased risk of infection [10].

Supporting this view, a recent case study demonstrated that robust immune responses were observed during clinical recovery from the COVID- 19 virus in a middle aged healthy adult [11,12]. Other studies also reported that the total number of NK and CD8^+^ T cells had decreased significantly in patients with SARS-CoV 2 infection [13] as a result of T cell infection by SARS-CoV 2 [14,15].

Recently, several studies have demonstrated that physical activity and meditation play a pivotal role in regulating inflammation and supporting immune function [16,17,18,19]. Consequently, general recommendations for a healthy lifestyle, including physical activities and meditation, have been made worldwide to help prevent disease, enhance immune function and improve global health and well-being [17,19]. Emerging evidence indicates that there are substantial benefits for practising Tai Chi and Qigong (TQ) for health and well-being. TQ, also known as moving meditation, is a classical mind-body exercise originating in China and has been utilised as a preventative health intervention for many centuries. TQ is the most common form of physical exercise among adults in China [20]. The 2012 US National Health Interview Survey (NHIS) data suggested that more than 7 million adults in the US practised TQ and its popularity is growing globally [21,22,23]. Reasons given for practicing TQ were to optimize overall health and well-being, prevent disease and prevent the progression of medical conditions [20,21]. Respondents who practice TQ indicated that the positive outcomes of practice were reduced levels of stress (83%) and improved overall health and well-being (74%) [21]. Recently, a number of systematic reviews and meta-analyses have demonstrated the positive impact of TQ on physical conditions such as arthritis [24], cancer [25], diabetes [26], falls prevention [27], fibromyalgia [28], osteoarthritis [29], chronic pain [24,30] and cognitive function [31]. Evidence also supports the psychological benefits of TQ, particularly for symptoms of anxiety and depression [32]. Furthermore, several descriptive reviews have examined the beneficial effects of mind-body interventions, i.e., TQ, yoga and meditation on immune function and immune system-related inflammatory biomarkers [33,34]. Another recent study conducted with healthy women demonstrated that TQ can change gene expression associated with inflammation (HSF1, HSPA1A, IL6, IL10, CCL2 and NF-kB mRNA) [35]. Although a number of studies have reviewed the effects of TQ on physical and psychological wellbeing [36,37], few studies have explored the effects of TQ on immunoregulatory responses. The aim of the current review is to assess the effects of TQ on immune function and immune system-related inflammatory biomarkers.

## 2. Methods

A systematic review and meta-analysis was conducted following the 2018 Preferred Reporting Items for Systematic Reviews and Meta-analyses (PRISMA) guidelines [38] for systematic reviews and meta-analyses. Electronic searches were conducted on four English databases (Medline, PubMed, Embase and ScienceDirect) from inception through to April 2020. Searches were performed using the following keywords: “Tai Chi or Qigong” and “immune system, immune function, immunity, Immun*, inflammation and cytokines”. Additional searches were performed in Google Scholar. Eligibility criteria were: full-text studies published in English, RCTs with the primary outcome of immune response, sample size (n ≥ 30), and a TQ intervention period of at least four weeks. Interventions using qigong (QG) or emitting qi therapy by a qi master were excluded. Purely meditational techniques, such as Zen meditation, were excluded. Two reviewers (KB and BO) screened the titles and abstracts and reviewed them for eligibility after reading the full-text. Additionally, searches were conducted for other potential studies by screening references in the identified studies.

### 2.1. Data Analysis

Outcomes at the initial post-intervention assessment were summarised and compared by TQ intervention arms. Estimates of intervention effects on immune responses (change of immune cells and inflammatory biomarkers) were extracted and compared for randomised arms [intervention, active control (exercise or health education) or non-active control (usual care/daily activities/wait-list)]. A random-effects meta-analysis was used to compute pooled estimates allowing for variation. 

Effect sizes were estimated from the difference between study group means divided by variances pooled from both treatment and control groups. Where necessary, known equations were used to calculate the effect sizes from the reported data [39,40]. Standardised mean differences (SMD, Hedge’s g) and 95% confidence intervals (CIs) were calculated. I^2^ was calculated to assess heterogeneity [40]. The outcomes of immune system and inflammatory-related biomarkers were reported in Table 1, and Figures 2–6. A negative SMD value indicated a greater decrease in biomarkers. 

### 2.2. Quality Assessment of Original Papers

Risk of Bias (RoB) Assessment: To adequately assess RoB of the included RCTs, two reviewers independently assessed the RoB using the Cochrane Collaboration’s tool for assessing RoB version 2 (RoB 2) [65]. The Cochrane Collaboration tool RoB2 consists of six domains: “randomization process”, “deviations from intended interventions”, “missing outcome data”, “measurement of the outcome”, “selection of the reported result”, and “overall bias”. Any disagreement between the two reviewers was resolved through discussion. 

## 3. Results

**Figure 1:**
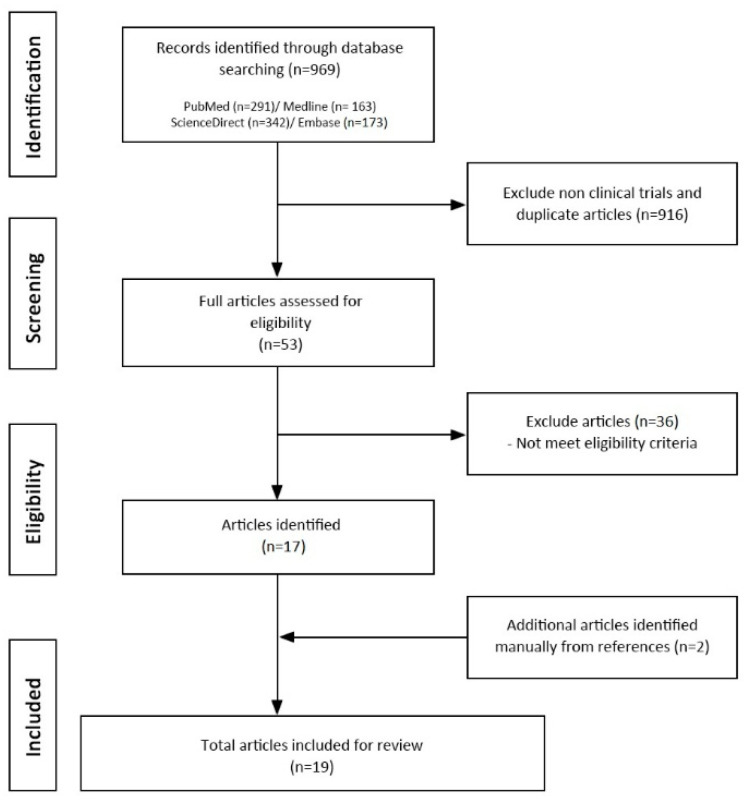
Flow chart.

A total of 969 studies were initially identified and screened in this literature search. After an in-depth evaluation of screening titles and abstracts, 53 articles remained for assessment of eligibility to be included in the review. Nineteen studies were included in the review (Figure 1). Seventeen studies were included in the meta-analyses.

### 3.1. Characteristics of Clinical Studies and Quality of Evidence

In the nineteen RCTs [Tai Chi (n = 14) and Qigong (n = 5)], there were a total of 1686 participants with an age range 18 to 87 years, and a sample size range of 32 to 252 participants within the studies, of which 775 were in the intervention groups and 911 in the control groups (Table 1). Studies were conducted across several countries viz. USA (n = 8), China (n = 4), Taiwan (n = 2), Australia (n = 1), Spain (n = 2), and one each were from Hong Kong and Thailand. Participants in the studies were categorised as cancer survivors (n = 5), older adults with a history of varicella (n = 2), healthy college students (n = 3), healthy older adults (n = 2), and a further seven studies with older adults with chronic neck pain (n = 1), mild cognitive impairment (n = 1), cardiovascular disease, (n = 1), diabetes (n = 1), insomnia (n = 1), HIV (n = 1), and depression (n = 1). Seventeen studies were designed with two arms while one study was conducted with three arms (TC vs. CBT vs. Health education) and one with four arms (TC vs. relaxation vs. spiritual growth vs. wait-list), respectively. In the control group conditions, physical exercise (n = 3), health education (n = 4) and/or CBT (n = 2), wait-list group (n = 2), and usual care and daily activities (n = 9) were used. The TQ intervention period varied from 4 weeks to 6 months and included periods of 4 weeks (n = 2), 8 weeks (n = 1), 10 weeks (n = 3), 12 weeks (n = 6), 15 weeks (n = 1), 16 weeks (n = 3) and 6 months (n = 3).The number of intervention sessions ranged from one to five times per week, with session frequencies of once per week (n = 5), two sessions per week (n = 3), three sessions per week (n = 9), and one study each with four sessions per week and five sessions per week, respectively. The majority of studies used an intervention time of 60 min (n = 7), whereas other intervention times comprised 30 to 50 min (n = 6), 90 min (n = 2), 120 min (n = 3) and 1 study did not report an intervention time. 

### 3.2. Outcomes on the Immune System and Inflammation Associated Biomarkers

**Figure 2:**
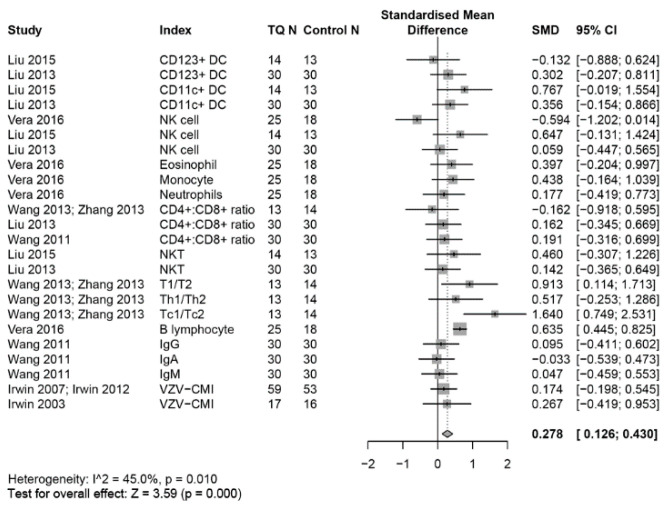
Forest plot for random-effects meta-analysis of the effects of TQ on the immune system.

**Figure 3:**
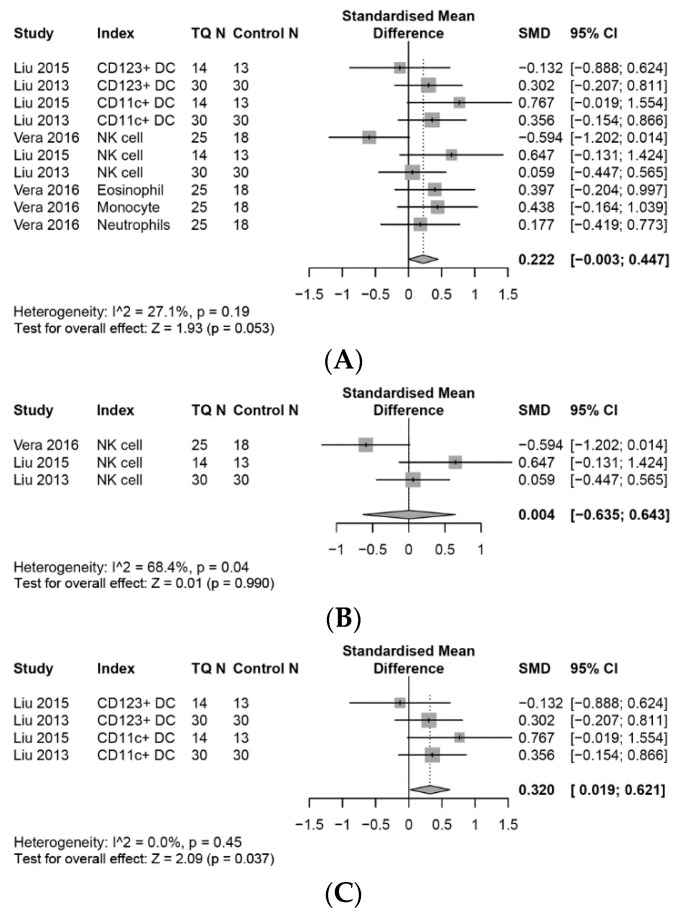
Forest plot for random-effects meta-analysis of the effects of TQ on the innate immune system. (**A**): The effects on the innate immune system, (**B**): the effects on the NK cells, (**C**): the effects on the DCs.

**Figure 4:**
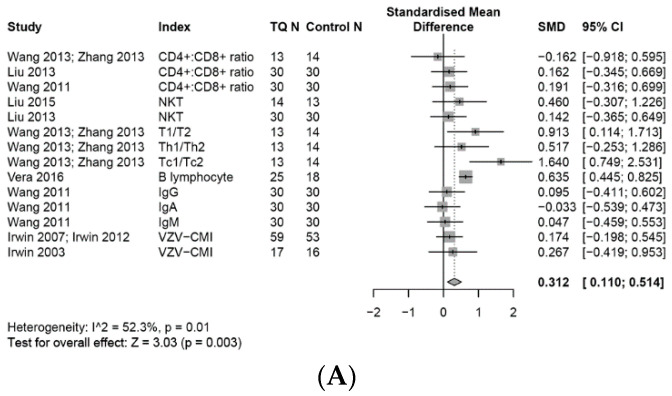

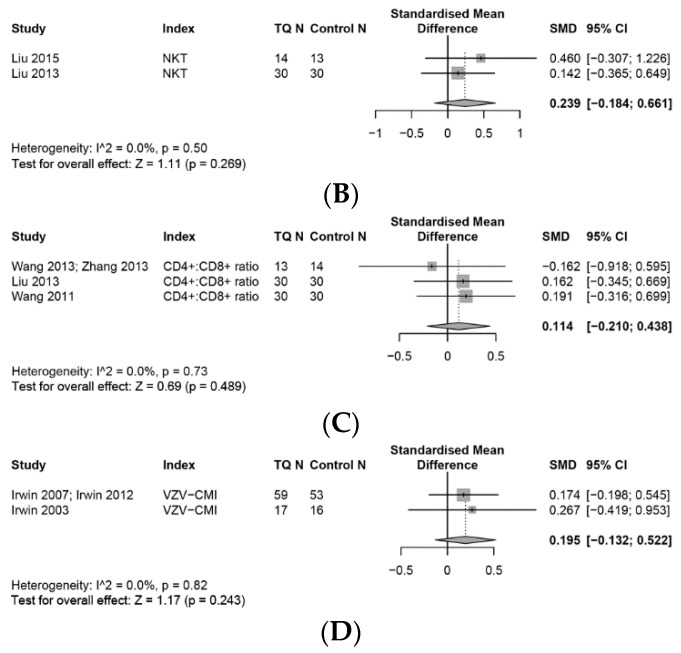
Forest plot for random-effects meta-analysis of the effects of TQ on the adaptive immune system. (**A**): The effects on the adaptive immune system, (**B**): the effects on the NKT cells, (**C**): the effects on the CD4+/CD8+ ratio, (**D**): the effects on the VZV-cell-mediated immunity.

**Figure 5:**
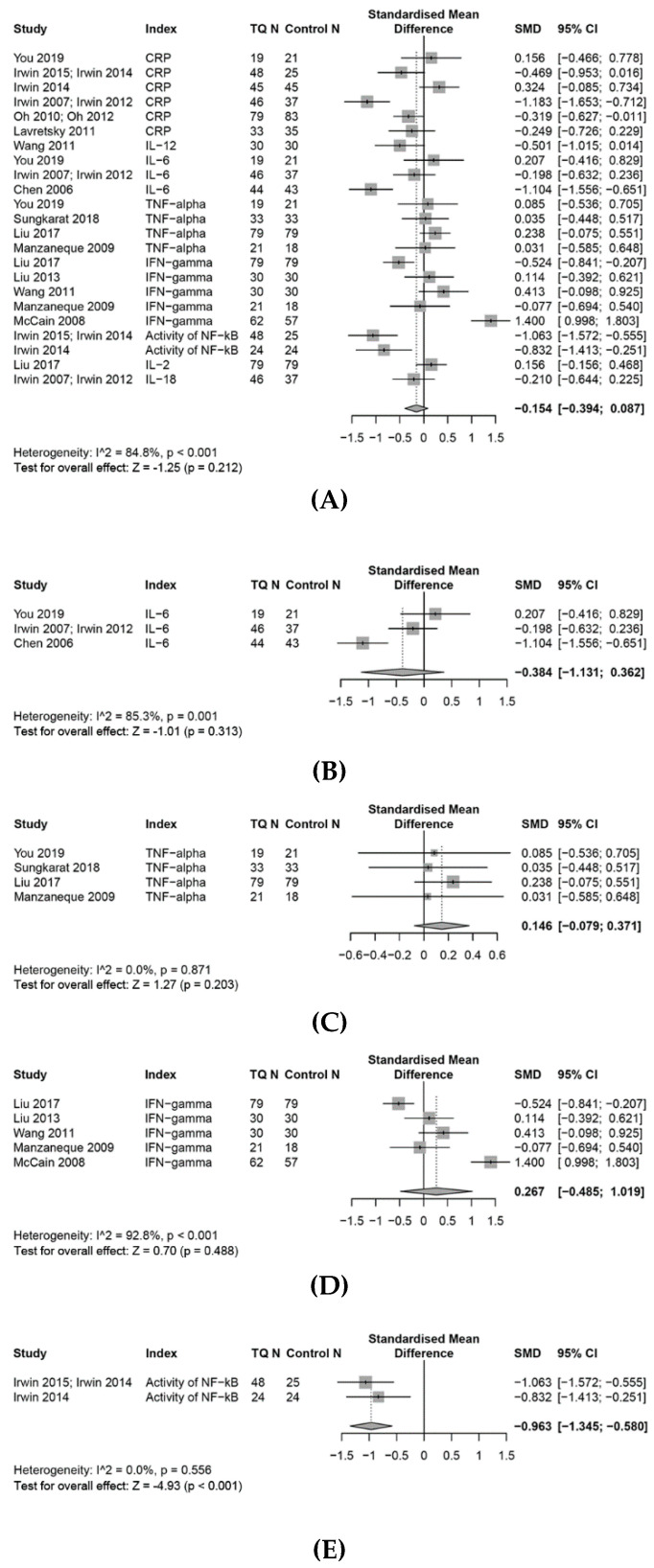
Forest plot for random-effects meta-analysis of the effects of TQ on the inflammation response. (**A**): The effects on the inflammation response, (**B**): the effects on the levels of IL6, (**C**): the effects on the levels of TNF-α, (**D**): the effects on the levels of IFN-γ, (**E**): the effects on the activity of NF-κB.

**Figure 6:**
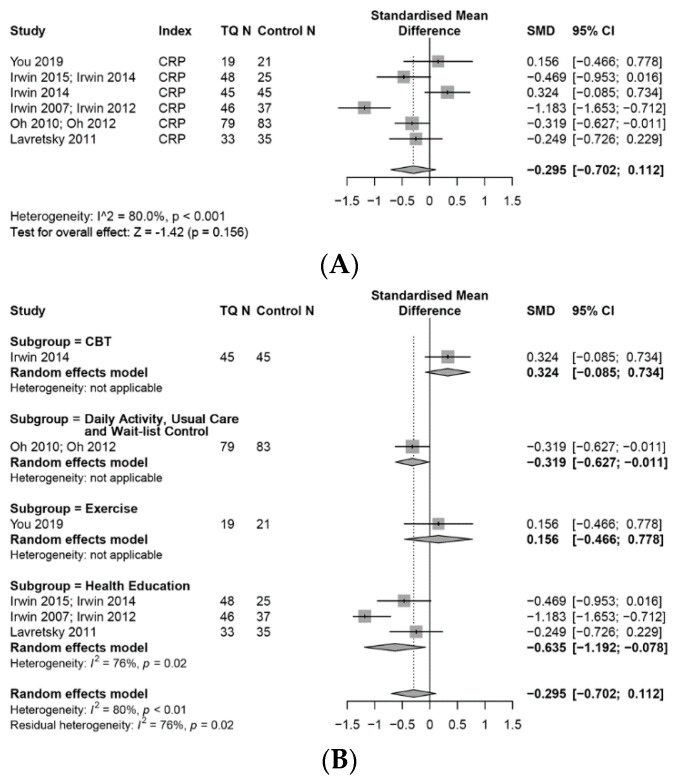
Forest plot for random-effects meta-analysis of the effects of TQ on levels of CRP. (**A**): The effects on the levels of CRP, (**B**): subgroup analysis of CRP based on control interventions.

The effects of TQ interventions on the selected immune system outcomes and inflammatory biomarkers are reported in Figure 2, Figure 3, Figure 4, Figure 5 and Figure 6. Meta-analysis data are presented as SMD (95%, CI) unless otherwise stated.

### 3.3. Outcomes on the Immune System

Overall, a random-effects meta-analysis found that TQ had a significant small effect of increasing the levels of immune cells (SMD, 0.28; 95% CI, 0.13 to 0.43, *p* < 0.01, I^2^ = 45%) (Figure 2).

### 3.4. Effects on the Innate Immune System 

Overall, a random-effects meta-analysis found that TQ had a small effect of increasing the levels of innate immune cells compared with controls (SMD, 0.22; 95% CI, −0.00 to 0.45, *p* = 0.05, I^2^ = 27%), with no significant heterogeneity across the studies (Figure 3A).

#### 3.4.1. NK Cells

A meta-analysis performed with three studies showed that there were no significant effects for the levels of NK cells (SMD, 0.00; 95% CI, −0.64 to 0.64, *p* = 0.99, I^2^ = 68%), (Figure 3B). Despite two studies reporting positive trends on NK cells, one study showed significant decreases in NK cells which may offset the effect size. 

#### 3.4.2. Dendritic Cells (DCs)

There were significant small effects on DCs (SMD, 0.32; 95% CI, 0.02 to 0.62, *p* = 0.04) in favour to TQ compared to control groups (Figure 3C).

### 3.5. Other Innate Immune Cells

A meta-analysis of one study which examined innate immune cells showed that TQ had a small effect of increasing the levels of eosinophils (SMD, 0.40; 95% CI, −0.20 to 1.00), monocytes (SMD, 0.44; 95% CI, −0.16 to 1.04), and a marginally small effect on neutrophils (SMD, 0.18; 95% CI, −0.42 to 0.77), compared with daily activities.

### 3.6. Effects on the Adaptive Immune System

#### 3.6.1. Adaptive Immune Cells 

Overall, a random-effects meta-analysis found that TQ had a small effect of increasing the levels of adaptive immune cells compared with controls (SMD, 0.31; 95% CI, 0.11 to 0.51, *p* = 0.01), I^2^ = 52%, with low to moderate heterogeneity across studies (Figure 4A–D).

#### 3.6.2. T Cell Associated Adaptive Immune Cells

A meta-analysis showed that TQ had a small but non-significant effect of increasing levels of NKT cells (SMD 0.24, 95% CI, −0.18 to 0.66, *p* = 0.27), a moderate effect on the Th1/Th2 ratio (SMD, 0.52; 95% CI, −0.25 to 1.29), and a significant large effect on the Tc1/Tc2 ratio (SMD, 1.64; 95 % CI, 0.75 to 2.53), compared with daily activities. Also, there was a small non-significant effect on the CD4^+^/CD8^+^ ratio (SMD, 0.11, 95% CI: −0.21 to 0.44, *p* = 0.49).

Other adaptive immune cell responses associated with the biomarker B lymphocytes showed a significant moderate effect for TQ increasing the proportion of B lymphocytes (SMD, 0.64; 95% CI, 0.45 to 0.83), but there were negligible effects for immunoglobulin antibodies IgA (SMD, −0.03; 95% CI, −0.54 to 0.47), IgG (SMD, 0.10; 95% CI, −0.41 to 0.60), and IgM (SMD, 0.05; 95% CI, −0.46 to 0.55), when compared with a health education control group. (Figure 4A–C). For VZV-cell-mediated immunity, two RCTs measured VZV responder cell frequency (VZV-RCF) compared with daily activity and health education controls. A meta-analysis found that TQ had a small effect (SMD, 0.20; 95% CI, −0.13 to 0.52), I^2^ = 0%, of elevating VZV-RCF (Figure 4D).

### 3.7. Effects on the Inflammation Response

Overall, a random-effects meta-analysis indicated that TQ had no significant effects on responses to inflammation (SMD, −0.15; 95% CI, −0.39 to 0.09, *p* = 0.21, I^2^ = 85%), as measured by the systemic inflammation biomarker CRP, the cell mediated biomarker cytokines (IL1β, IL2, IL4, IL6, IL10, IL12, IL18, TNF-α, INF-γ, GCSF) and NF-κB, compared with controls, due to several bidirectional cytokine responses. However, sub-group analyses showed TQ had positive trend on levels of CRP, IL6 and NF-κB, separately (Figure 5A–E).

#### 3.7.1. CRP 

A meta-analysis conducted with six studies suggested that TQ had a small effect of reducing CRP compared with control groups (SMD, −0.30; 95% CI, −0.70 to 0.11, *p* = 0.16, I^2^ = 80%). Furthermore, a subgroup analysis (Figure 6A,B) of the effect of TQ on CRP, with different control conditions (TQ vs. health education control, TQ vs. exercise control, TQ vs. inactive control, TQ vs. CBT), showed that studies that compared TQ with “health education” resulted in a moderate effect (SMD −0.64, 95% CI, −1.19 to −0.08, I^2^ = 76%), of reducing CRP, and with “inactive (usual care)” resulted in a small effect (SMD, −0.32; 95% CI, −0.63 to −0.01) of reducing CRP. In contrast, a study that compared TQ with an “exercise” group, demonstrated non-significant effects (SMD, 0.16; 95% CI, −0.47 to 0.78), and another study comparing TQ with “Cognitive Behavioural Therapy (CBT)” showed a small effect of increasing CRP (SMD, 0.32; 95% CI, −0.09 to 0.73). 

#### 3.7.2. IL-6

A meta-analysis of three studies suggested that, compared to controls, TQ had a small effect of reducing the levels of IL-6 (SMD, −0.38; 95% CI, −0.13 to 0.36, I^2^ = 85%). Of these three studies, one study showed that TQ increased the levels of IL-6, while another two studies showed reduced IL-6 levels following a TQ intervention.

#### 3.7.3. TNF-α

A meta-analysis of four RCTs found that TQ had a negligible effect on levels of TNF-α compared with controls (SMD, 0.15; 95% CI, −0.08 to 0.37, I^2^ = 0%). 

#### 3.7.4. INF-γ

A meta-analysis of five RCTs showed that TQ had a small effect of increasing the level of INF-γ (SMD, 0.27; 95% CI, −0.49 to 1.02, I^2^ = 93%). This result may have been influenced by considerable heterogeneity. 

#### 3.7.5. Effects on Other Pro-Inflammatory Biomarkers

A meta-analysis conducted on one study showed a non-significant effect of TQ on levels of IL-2 (SMD, 0.16; 95% CI, −0.16 to 0.47) when compared with controls, whereas another study showed a small effect of TQ on levels of IL-18 (SMD, −0.21; 95% CI, −0.64 to 0.23), and a third study showed a moderate effect for TQ on IL-12 (SMD, −0.50; 95% CI, −1.02 to 0.01). Another meta-analysis found larger effects in favour to TQ on levels of NF-κB (SMD, −0.96; 95% CI, −1.35 to −0.58). 

### 3.8. Effects on Anti-Inflammatory Biomarkers

#### IL-4 

A meta-analysis conducted on two studies suggests that TQ had no significant effect on levels of IL-4 (SMD, 0.03; 95% CI, −0.33 to 0.39, I^2^ = 0%), when compared with controls (data not presented). 

### 3.9. Assessment of Risk of Bias 

**Figure 7:**
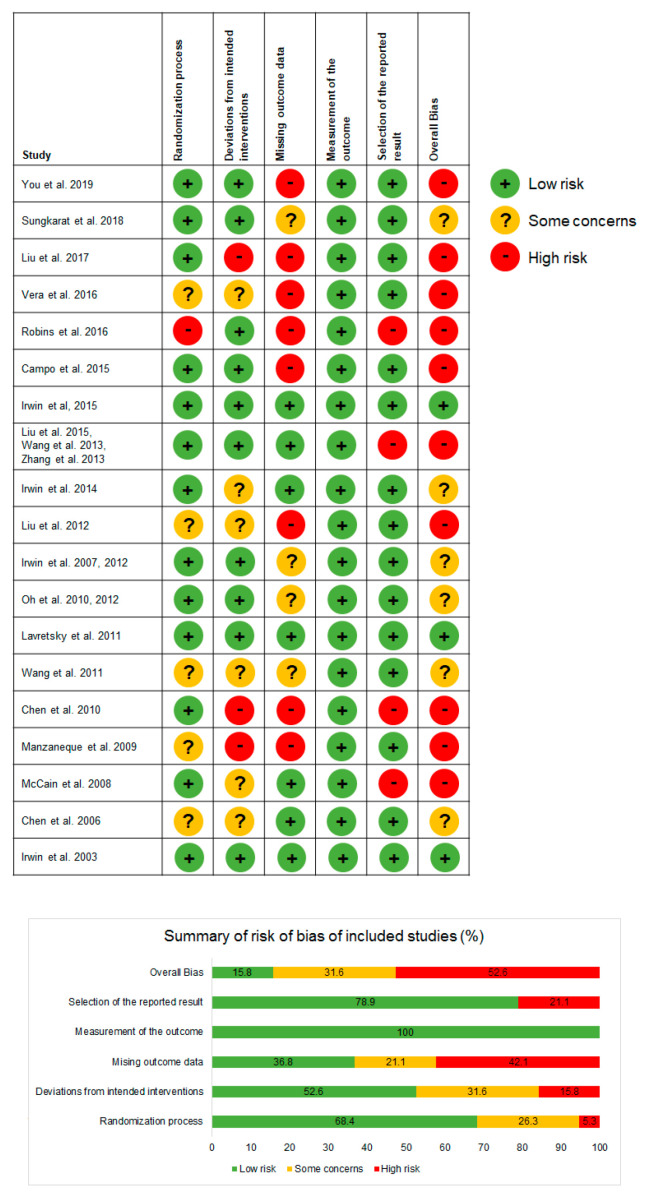
Risk of bias assessment.

A RoB assessment was conducted with a revised tool (RoB 2) [65], to examine randomization process, deviations from intended interventions, missing outcome data, measurement of the outcome, selection of the reported result, and overall bias. In an overall assessment of bias, three RCTs were assessed as having a low RoB, six RCTs with some RoB concerns and ten RCTs with high RoB. In the domain of “measurement of the outcome” all of the reviewed RCTs have low RoB, whereas in domain of “missing outcome data” the majority of reviewed studies displayed high RoB. Individual scores for RoB are presented in Figure 7.

## 4. Discussion

In this systematic review and meta-analysis of nineteen RCTs that examined the effects of TQ on the immune system and inflammation, we found that TQ is capable of modulating immune system functioning and inflammatory biomarker responses. An important finding of the current review was that a minimum of 4 weeks practice of TQ enables participants to enhance their immune system functioning by stimulating innate and adaptive immune cell responses and regulating biomarkers associated with inflammation. In addition, we found two studies that showed that practising TQ for more than 12 weeks can alter gene expression, as demonstrated in NF-κB signal pathways. 

Our findings are comparable with similar studies that assessed the effect of mind-body therapies using mixed interventions including meditation, Yoga and TQ [33,66]. Prior reviews that examined the effect of TQ on immunity and inflammation have reported no strong evidence of a favorable effect of TQ on inflammation [67] and the immune system [68] and insufficient evidence to support the clinical application of TQ to reduce infection [68,69]. These earlier reviews included fewer RCTs, and non-RCT studies, and a less comprehensive literature search which limits the conclusions made. 

Another important aspect of the present review is that, to the best of our knowledge, this is the first comprehensive systematic review specifically evaluating the effects of TQ on the immune system and inflammatory biomarker responses. In order to assess the effect of TQ on the immune system and inflammation, immune related cell types were categorized into two groups, viz. innate immune cells and adaptive immune cells, and inflammatory biomarkers into three groups, viz. the systemic inflammatory biomarker CRP, cytokines and gene expression associated with pro-inflammatory processes. Of the nineteen reviewed RCTs, four studies measured innate immune cells (eosinophils, monocytes, neutrophils, NK and dendritic cells), six studies measured adaptive immune cells (T cells, NKT, and B cells), and four studies measured both. Overall, a random-effects meta-analysis found that TQ had a significant small effect of increasing the levels of immune cells (SMD, 0.28; 95% CI, 0.13 to 0.43, *p* < 0.01, I^2^ = 45%), compared to controls. The effect of TQ on inflammation is measured commonly by levels of the inflammatory biomarkers CRP, IL6 and TNF-α. In general, the levels of CRP decreased following a TQ intervention. Of the eight studies measuring CRP, four studies reported that TQ significantly decreased levels of CRP compared to controls, whereas three studies conducted with older adults with symptoms of chronic ill-health (pain, insomnia and older adults with history of varicella) showed no differences between the intervention and control groups (Table 1). Considering that CRP is a commonly used diagnostic marker of systemic inflammation, non-significant changes in levels of CRP may be associated with the progression of the chronic disease in these older participants. Given these mixed results, further investigation of this aspect, with a homogeneous study population, is warranted. In addition to CRP, cell mediated inflammatory cytokines (IL6, TNF-α) were also found to demonstrate an overall trend of reduced levels following the TQ intervention. However, for inflammatory cytokines (IL2, IL4, IL6, IL10, IL12, TNF-α, INF-γ) both downregulation and upregulation of cytokine responses were observed across the studies. 

These bidirectional results for inflammatory cytokines are consistent with the results of recent studies that examined cytokine levels following mind-body therapies [33,66] and exercise [70] interventions, that also included TQ. Several studies have suggested that increases in inflammatory cytokines are not only in response to immune system activation or infection, but also can occur when immune cells are stimulated to activate cytotoxicity [71,72]. For example, a review paper on pro-inflammatory and anti-inflammatory processes in patients with multiple myeloma, examined the effects of locally produced cytokines, as a primary immune response, and found that efficacious tumour immunosurveillance due to tumour-specific CD4^+^ T cells was consistently related to increased local concentrations of both proinflammatory (IL-6, IL-1α, and IL-1*β*) and Th1-associated cytokines (IL-2, IL-12, and IFN-*γ*) [71]. It was concluded that the influence of cytokines on the immune system occurs as parallel processes and that changes in one specific cytokine can be balanced by others within the cytokine system, leading to a modulation of the immune response. In light of the current findings of a bidirectional response of inflammatory cytokines, additional clinical trials with a homogeneous study population will help to better understand the directional nature of the relationship between inflammation and immunity. Despite these mixed results in outcomes for inflammatory responses, as measured by levels of CRP, cytokines, and NF-κB, overall, there were trends towards reduced levels of inflammation compared with control conditions.

Several limitations were identified in the current study. Firstly, caution is required in interpreting the overall random effect size on immune cells (SMD, 0.28; 95% CI, 0.13 to 0.43) and inflammation responses (SMD, −0.15; 95% CI, −0.39 to 0.09). Studies included in this review measured changes in a range of immune system and inflammatory biomarkers, rather than changes in a single identical biomarker in each study, thus confounding assumptions for independent variables associated with confidence intervals and heterogeneity in the meta-analysis. 

Secondly, the demographic profile of participants in the original studies included in this review were heterogeneous in respect of age, ranging from 18 to 89 years, and health status viz. from healthy to various symptoms of medical conditions, which limit the generalizability of our findings. Furthermore, the TQ interventions that were examined were heterogeneous in respect of duration (from 4 weeks to 6 months), frequency (one to five times per week) and type of TQ intervention. Considering the heterogeneity of these studies, future investigations into the modulatory effects on immune responses of different types of TQ interventions and their dosage levels will be worthwhile. For example, one study was conducted with a TQ intervention duration of 4 weeks and a frequency of three sessions per week, whereas the duration of other studies varied from 4 weeks to 24 weeks with frequencies ranging from one session to five sessions per week. However, at present there is no standardised protocol to inform healthcare professionals and the general public on the minimum dosage levels of TQ required to modulate immune responses [73]. Finally, the current review did not investigate the physiological mechanisms underlying the effects of TQ on immune responses, despite previous studies attempting to explain these potential mechanisms in mind-body medicine and psychoneuroimmunology models [33]. Given the complexity of TQ as a movement-based mind-body therapy, compared with other mind-body therapies that have less movement components, investigating these underlying TQ mechanisms will provide important insights into future clinical applications. Moreover, some recent RCTs comparing TQ with exercise or CBT have suggested that there were no significant differences in outcomes between the intervention groups, indicating that TQ may be equivalent to conventional exercise or CBT interventions. However, more studies with robust study designs and adequate statistical power are required. Considering that the immune system is vital for protection from external pathogens, including bacterial and viral pathogens often occurring in the natural environment, the evidence supporting TQ for a healthier immune system, can have important implications for promoting TQ programs for health and well-being. Recommending TQ programs for people with low immunity, particularly those receiving treatment that induces immune suppression and older adults with chronic diseases, could have a beneficial effect of strengthening immune function. More clinical outcome studies are required to examine TQ as a stand-alone or adjunctive intervention for these patients and its effects on comparative rates of recovery. For the general public, TQ offers a preventative health measure that can strengthen the immune system and assist overall health and well-being. 

In conclusion, despite several limitations, the current review of RCTs indicates that practising TQ can have a positive impact on immune system functioning and inflammatory processes. Given the vital role of the immune system, and in particular the influence of cytokines, in the current viral pandemic, preventative public health strategies to improve immune functioning in the general public, and those with medical conditions, are needed. However, while the promotion of TQ to the general public and healthcare professionals as a preventative health intervention for strengthening the immune system is recommended, further robust studies to develop clinical practice guidelines for using TQ with vulnerable populations is warranted.

## Figures and Tables

**Table 1:** Study characteristics of the 19 randomized clinical trials.

Author/Years/Reference	Population	Sample Size (% Female)	Mean Age (Range)	Intervention Duration, Type	Intervention Frequency, Style	Control Condition	Results ↑ ↓
You et al. [41] 2019 China	Older adults with chronic pain	N = 40 (43%) TC (n = 19) CG (n = 21)	74 (65–87)	12 weeks; TC	60 min, 2 times/week, 8-form Yang style	Light physical exercise	_NS_ CRP _NS_ IL-6 _NS_ TNF-α
Sungkarat et al. [42] 2018 Thailand	Older adults with mild cognitive impairment	N = 56 (86%) TC (n = 29) CG (n = 27)	68	6 months; TC	50 min, 3 times/week, TC in-class for 9 sessions (3 times per week for 3 weeks) plus practice at home 3 times a week with video, 10 forms TC	Health Education	_NS_ TNF-α _NS_ IL-10
Liu et al. [43] 2017 Hong Kong	Women with breast cancer	N = 158 (100%) QG (n = 50) CG (n = 51)	51 (21–80)	24 weeks; QG	60 min session, 2 times/week for 24 weeks, Guolin-Qigong	Stretching exercise	_NS_ IL-2 _NS_ IFN-γ ↑ TNF-α: (95% CI, 0.48–3.41, *p* < 0.03)
Vera et al. [44] 2016 Spain	College students	N = 43 (79%) QQ (n = 23) CG (n = 18)	(18–21)	4 weeks; QG	25–30 min, 3 times/week In total 20 qigong sessions, Taoist QG	Daily activities	↑ Number of B lymphocytes (*p* = 0.006) ↑ % of B lymphocytes (*p* = 0.006) ↓ % NK (*p* = 0.05) _NS_ Number of NK _NS_ Neutrophils, _NS_ Monocytes, _NS_ Eosinophils,
Robins et al. [45] 2016 USA	Women with high risk of cardiovascular disease	N = 96 (100%) TC (n = 47) CG (n = 49)	43 (35–50)	8 weeks;TC	60 min, 1/week, TC (short form)	Wait-list control	↓ TNF-α (*p* < 0.002) ↓ IFN-γ (*p* < 0.002) ↓ IL 8 (*p* < 0.026) ↓ IL-4 (*p* < 0.001) Result not reported (IL-1β, IL-2, IL-10, IL-12, IL-6, GCSF, CRP)
Campo et al. [46] 2015 USA	Older female cancer survivors	N = 63 (100%) TC (n = 29) CG (n = 25)	67	12 weeks; TC	60 min, 3 times/week, TC (19 movements)	Health education	_NS_ IL-6 _NS_ IL-12 _NS_ TNF-α _NS_ IL-4 _NS_ IL-10
Irwin et al. [47,48] 2015 and 2014 USA	Older adults with insomnia	N = 123 (73%) TC (n = 48) CBT (n = 50) CG (n = 25)	65 (55–85)	16 weeks; TC	120 min, 1/week, TC	Health education, CBT	↓ CRP (n = 0.06) ↓ % monocytes producing ↓ IL-6 (*p* < 0.01) ↓ % monocytes producing TNF-α (*p* < 0.01) ↓ % monocytes co-producing TNF-α and IL-6 (*p* < 0.01) at different time points ↓ Pro-inflammatory gene expression (NF-kB, IRF+, AP-1) ((month 4, *p* < 0.001).
Liu et al. [49] 2015 Wang et al. [50] 2013 Zhang et al. [51] 2013 China	Lung cancer survivors	N = 32 (50%) TC (n = 16) CG (n = 16)	61	16-weeks; TC	60 min, 3 times/week, Yang style 24-form	Daily activities	↑ NKT cells (*p* < 0.05) ↑ % NKT cells (*p* < 0.05) _NS_ NK cells _NS_ % NK cells _NS_ DC 123c cells _NS_ % of DC 123c cells ↑ DC11c cells (*p* < 0.01) _NS_ % DC11c cells T lymphocyte cells (T1) and (T2) ↑ T1/T2 ratio (*p* < 0.01) ↑ Tc1/Tc2 ratios (*p* < 0.01) _NS_ TH1/TH2 ratio ↓ T2 (*p* < 0.001) ↓ Tc2 (*p* < 0.003) ↑ Th2 (*p* < 0.025) ↓ CD55 (*p* < 0.05) _NS_ T-helper/T-suppressor (CD4^+^:CD8^+^ ratio) _NS_ CD59 expression
Irwin et al. [52] 2014 USA	Breast cancer survivors with insomnia	N = 90 (100%) TC (n = 45) CG (n = 45)	59 (42–83)	12 weeks; TC	120 min, 1/week, TC	CBT	_NS_ CRP ↓ % monocytes producing IL-6 (*p* = 0.07) ↓ % monocytes producing TNF-α (*p* < 0.05) ↓ % monocytes coproducing TNF-α and IL-6 (*p* < 0.02) ↓ Proinflammatory mediators (NF-kB) (*p* = 0.001)
Liu et al. [53] 2012 China	Healthy middle-aged and older women	N = 60 TC (n = 30) CG (n = 30)	54 (50–65)	6 months; TC (24 forms and 42 Sword forms)	60 min, 4 times/week TC (24 forms and 42 Sword forms)	Daily activities	↑ % of CD4+ T lymphocytes (*p* < 0.05) _NS_ CD3 _NS_ CD8+ ↑ CD4+:CD8+ ratio (*p* < 0.05) ↑ % of NK cell (*p* < 0.05) ↑ % of NKT cells (*p* < 0.05) ↑ TNF- γ (*p* < 0.05) _NS_ IL-4 ↑ % of CD123+ DCs (*p* < 0.01) ↑ % of CD11c+ DCs (*p* < 0.01)
Irwin et al. [54,55] 2007 and 2012 USA	Healthy older adults with history of varicella	N = 112 (61%) TC (n = 59) CG (n = 53)	70 (59–86)	16 weeks; TC	40 min, 3 times/week for a total of 120 min, TC with 20 movements	Health education	↑ Levels of VZV-CMI (*p* < 0.05) _NS_ IL-6 (*p* = 0.06) _NS_ IL-18 _NS_ CRP _NS_ sIL-1RA _NS_ sIL-6R _NS_ sICAM,
Oh et al. [56,57] 2012 and 2012 Australia	Cancer survivors	N = 162 (57%) TC (n = 79) CG (n = 83)	60 (31–86)	10 weeks; QG	90 min, 2 times/week + individual practice recommended for 30 min per day, Medical QG	Usual Care	↓ CRP (*p* < 0.044)
Lavretsky et al. [58] 2011 USA	Older adults with major depression	N = 73 (62%) TC (n = 36) CG (n = 37)	71	10 weeks; TC	120 min, 1 times/week TC Chih	Health education	↓ CRP (*p* < 0.05)
Wang et al. [59] 2011, China	Healthy sedentary female college students	N = 60 (100%) TC (n = 30) CG (n = 30)	19	12 weeks; TC	45 min, 5 times/week; TC 24 standardized movements	Daily activities	_NS_ IgG _NS_ IgA _NS_ IgM _NS_ (CD3, CD4+, CD8+) _NS_ IFN-γ _NS_ IL-4 _NS_ IL-12
Chen et al. [60] 2010 Taiwan	Adults with diagnosis of Type II diabetes and BMI 30–35	N = 104 (43%) TC (n = 56) CG (n = 48)	58	12 weeks; TC	60 min, 3 times/week; Chen-style 99-form	Conventional aerobic exercise	↓CRP (*p* < 0.014)
Manzaneque et al. [61] 2009 Spain	Healthy college students	N = 39 (87%) QG (n = 21) CG (n = 18)	18–21	4 weeks; QG	30 min, 3 times/week; Ba Duan Jin Qg	Daily activities	_NS_ TNF-α _NS_ IFN-γ
McCain et al. [62] 2008 USA	Adults with diagnosis of HIV	N = 252 (40%) TC (n = 62) RLXN (n = 65) SPRT (n = 68) CG (n = 57)	42	10 weeks; TC	90 min, 1 time/week; Focused short form TC training with 8 movements	Wait-list control	_NS_ CD4+, CD8+, and CD57+ T lymphocytes: _NS_ NKC cytotoxicity _NS_ Lymphocyte proliferation _NS_ TNF-α _NS_ IFN-γ _NS_ IL-2 _NS_ IL-4 _NS_ IL-6 _NS_ IL-10
Chen et al. [63] 2006 Taiwan	Healthy middle-aged women with BMD T scores ≥ −2.5	N = 87 (100%) QG (n = 44) CG (n = 43)	45 (35–60)	12 weeks; QG	3 times/week; Ba Duan Jin Qg 8 sections	Daily activities	↓ IL-6 (*p* < 0.001)
Irwin et al. [64] 2003 USA	Healthy older adults with history of varicella	N = 36 (72%) TC (n = 18) CG (n = 18)	70	15 weeks; TC	45 min, 3 times/week for a total of 45 sessions; TC with 20 standardized movements	Wait-list control (maintenance of routine activities	↑ Levels of VZV-CMI (*p* < 0.05)

Abbreviations: IL-2: interleukin-2, IFN-γ: interferon-γ, TNF-α: tumor necrosis factor-α, CRP: C-reactive protein, IL-1: interleukin-1, IL-4: interleukin-4, IL6: interleukin-6, IL-10: interleukin-10, IL-18: interleukin-18, sIL-1RA: soluble IL-1 receptor antagonist, sIL-6R: soluble IL-6 receptor, sICAM: soluble intercellular adhesion molecule, VZV-RCF: varicella zoster virus responder cell frequency, IgG: Immunoglobulin G, IgA: Immunoglobulin A, IgM: Immunoglobulin M, NKC: natural killer cell; NKT: natural killer T cell, DC: Dendritic cells, CRP:C-reactive protein, IL-1ra: interleukin 1 receptor antagonist, RLXN: relaxation training, SPRT: spiritual growth groups, CBT: Cognitive Behavioural Therapy, GLQG: Guolin Qigong, TC: Tai Chi, QG: Qigong, TQ: Tai Chi and Qigong, CG: Control group, NS: Not significant, ↑: increase, ↓: decrease.
